# Single-molecule imaging reveals modulation of cell wall synthesis dynamics in live bacterial cells

**DOI:** 10.1038/ncomms13170

**Published:** 2016-10-24

**Authors:** Timothy K. Lee, Kevin Meng, Handuo Shi, Kerwyn Casey Huang

**Affiliations:** 1 Department of Bioengineering, Stanford University, Stanford, California 94305, USA; 2 Program in Biomedical Informatics, Stanford University, Stanford, California 94305, USA; 3 Department of Microbiology and Immunology, Stanford University School of Medicine, Stanford, California 94305, USA

## Abstract

The peptidoglycan cell wall is an integral organelle critical for bacterial cell shape and stability. Proper cell wall construction requires the interaction of synthesis enzymes and the cytoskeleton, but it is unclear how the activities of individual proteins are coordinated to preserve the morphology and integrity of the cell wall during growth. To elucidate this coordination, we used single-molecule imaging to follow the behaviours of the two major peptidoglycan synthases in live, elongating *Escherichia coli* cells and after perturbation. We observed heterogeneous localization dynamics of penicillin-binding protein (PBP) 1A, the synthase predominantly associated with cell wall elongation, with individual PBP1A molecules distributed between mobile and immobile populations. Perturbations to PBP1A activity, either directly through antibiotics or indirectly through PBP1A's interaction with its lipoprotein activator or other synthases, shifted the fraction of mobile molecules. Our results suggest that multiple levels of regulation control the activity of enzymes to coordinate peptidoglycan synthesis.

## Abstract (web summary)

The bacterial cell wall is important for cell shape and stability, but how the activities of the biosynthetic machinery are coordinated are not clear. Here the authors use single-molecule imaging and chemical perturbations to determine factors that affect the localization dynamics of penicillin-binding proteins (PBP)1A and PBP1B.

The cell wall is a macromolecular network of glycan strands cross-linked by short peptides, and its synthesis in Gram-negative bacteria such as *Escherichia coli* is subject to regulation by proteins located throughout the cell envelope^1^,^2^. Peptidoglycan subunits are synthesized in the cytoplasm^3^,^4^, flipped across the membrane^5^, and incorporated into the existing wall by a host of enzymes, including the PBPs^6^. Several of the PBPs are redundant, and often phenotypes arise only when multiple enzymes are perturbed^7^. Decoupling the activities of multiple enzymes with redundancy requires quantitative phenotypic analyses and systematic perturbations; PBPs are an excellent case study, particularly in *E. coli*, given the accessibility of antibiotics that specifically target individual enzymes^8^ and even specific enzymatic functions^9^.

PBP1A and 1B are bifunctional enzymes with both glycosyltransferase (strand polymerization) and transpeptidase (crosslinking) activities^10^. Immunofluorescence localization showed that PBP1A is involved primarily in cell elongation, while PBP1B participates mostly in cell division^11^,^12^. The outer-membrane lipoproteins LpoA/B were identified as co-factors that activate PBP1A/B, respectively^13^,^14^. *In vitro*, LpoA activates the transpeptidase activity of PBP1A, and LpoB activates the glycosyltransferase activity of PBP1B, although the stimulation of one activity also increases the other^15^. PBP2 is an essential transpeptidase^16^ that has been shown to bind PBP1A and enhance its transpeptidase activity *in vitro*^11^.

MreB is an actin-like protein^17^ that is essential for rod-shaped growth^18^. Clusters of MreB move along approximately linear tracks in a circumferential manner that dictates the spatial pattern of cell wall synthesis^19^,^20^,^21^,^22^. Although PBP2 activity is required for processive MreB motion, single-particle tracking photoactivated localization microscopy (sptPALM) revealed that PBP2 undergoes diffusive rather than ballistic motion^23^, challenging the notion of a processive, stable complex of PBPs moving along with MreB. This diffusive motion buffers growth rate in the presence of fluctuations in the concentration of an essential protein^23^, and suggests that such distributed activity may be generally beneficial. However, it is still unknown whether other members of the synthesis machinery move with MreB, move diffusively but in a complex with PBP2, or diffuse independently.

Using single-molecule imaging, we find that PBP 1A, 1B and 2 diffuse at different rates in the inner membrane. In addition, PBP1A motion is reduced on perturbation to its enzymatic activity and is further modulated by interactions with low molecular weight PBPs. We also find that PBP1A dynamics depend on its cognate lipoprotein activator, LpoA, consistent with its role in regulating PBP1A biochemical activity. Our single-molecule analyses indicate that PBP1A dynamics are heterogeneous and that perturbations alter the proportion of mobile PBP1A molecules. These results suggest that multiple levels of regulation control the activity of PBP1A during cell elongation and provide a framework for elucidating other interactions in the peptidoglycan synthesis network.

## Results

### PBPs exhibit different diffusion constants

**Figure 1:**
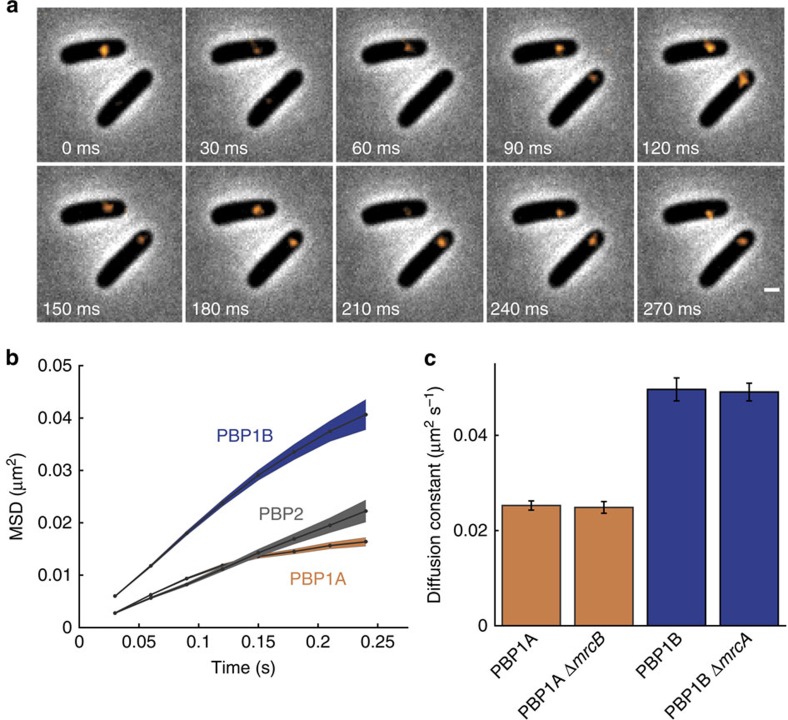
PBP1A and PBP1B diffuse at different rates. (**a**) TIRF images of PAmCherry-PBP1A molecules (orange) imaged every 30 ms in live *E. coli* (TKL241) at 30 °C overlaid on phase-contrast images. Scale bar: 1 μm. (**b**) MSD of PBP1A (*n*=3,177 molecules, TKL241), PBP1B (*n*=898 molecules, TKL211), and PBP2 (*n*=716 molecules, TKL130) fused to PAmCherry. Shaded area represents s.e.m. (**c**) Apparent diffusion constants calculated from a linear fit to the MSD for PBP1A in wild-type (*n*=3,177 molecules, TKL241) and Δ*mrcB* (lacking PBP1B; *n*=1,994 molecules, TKL240) backgrounds and for PBP1B in wild-type (*n*=898 molecules, TKL211) and Δ*mrcA* (lacking PBP1A; *n*=1,491 molecules, TKL239) backgrounds. Error bars indicate the s.d. of 1,000 bootstrap samples.

To determine whether PBP1A and PBP1B exhibit directed motion (mean-squared displacement (MSD) ∝ *t*^2^), similar to MreB, or diffusive motion (MSD∝*t*), similar to PBP2 (ref. 23), we fused each protein individually to photoactivatable mCherry (PAmCherry) and integrated these fusions into the chromosome at the native locus with expression from the native promoter to mimic physiological levels as closely as possible (Methods section). We then tracked the motion of single molecules in elongating, exponentially growing cells using sptPALM^23^; we verified that cells maintained their elongation rate during the short (∼15 s) period of illumination with the 405-nm activation laser (Supplementary Fig. 1, Methods section). PAmCherry-PBP1A/B molecules in live *E. coli* cells growing at 30 °C were imaged with total internal reflection fluorescence (TIRF) microscopy every 30 ms (Fig. 1a). Tracking measurements were restricted to the TIRF field, which excludes most of the poles and the division site once constriction has progressed by more than ∼100–150 nm. The MSD (Fig. 1b) calculated from single-molecule tracks suggests that the movements of PBP1A and PBP1B are diffusive, with a linear dependence versus time, rather than directed, which would result in a quadratic dependence. Furthermore, the relative slopes of the MSD traces indicates that the diffusion of PBP1B is significantly higher than that of PBP1A or PBP2 (*P*<0.0001, permutation test). The diffusion constants of both proteins are substantially below previous measurements for membrane proteins of similar size reconstituted in vesicles (∼2-5 μm^2^ s^−1^) (ref. 24), indicating that other interactions act to reduce the effective diffusion constants; a recent study observed that diffusion of a set of membrane proteins in *E. coli* cells was MreB-dependent and in the range 0.026–0.21 μm^2^ s^−1^ (ref. 25). The PBP1A MSD saturated after 0.2–0.3 s (Fig. 1b), suggesting subdiffusive behaviour or multiple populations of distinct diffusive behaviours. These measurements imply that PBP1A and PBP1B, similar to PBP2, are not stably associated with an MreB complex, but move at different rates. Nonetheless, treatment with A22, a small molecule that rapidly depolymerizes MreB^26^, led to a small, but significant, increase in the diffusion constant (Supplementary Fig. 2), providing further support for the role of MreB in organizing cell wall synthesis. Chloramphenicol treatment also increased the diffusion of PBP1A (Supplementary Fig. 2), suggesting that faster motion is correlated with a state of less cell-wall insertion by PBP1A.

This difference in diffusion constants between PBP1A and PBP1B would not be expected on the basis of protein size alone, since these proteins have very similar molecular weights. PBP1A/B are synthetic lethals, indicating that their collective activity is essential for cell growth^27^; cells expressing a PAmCherry-PBP1A fusion in the presence of a deletion for PBP1B (Δ*mrcB*) grew similarly to wild-type cells (Supplementary Fig. 3), indicating that the fusion is functional. To ensure that the activity of one of PBP1A/B is not obscuring the dynamics of the other, we determined that the dynamics of these proteins do not change when one protein is deleted (Fig. 1c and Supplementary Fig. 4a). This experiment also demonstrated the functionality of our fusions, since either PBP1A or PBP1B is required for viability, and these strains maintained a rod-like cell shape (Supplementary Fig. 4b). We hypothesized that the differential dynamics are related to their functional roles *in vivo* and that these dynamics could be modulated by perturbations to their enzymatic activities.

### Inhibition with certain antibiotics reduces PBP1A diffusion

**Figure 2:**
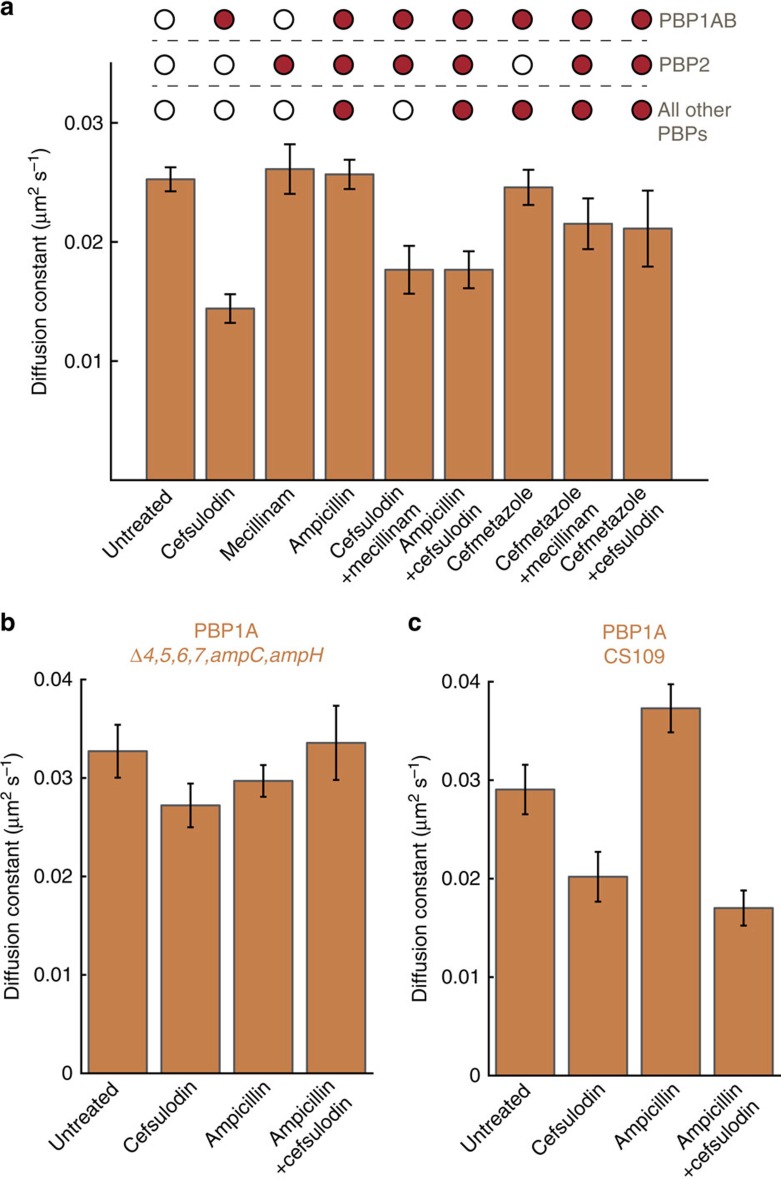
PBP1A diffusion depends on its enzymatic activity and interactions with other PBPs. (**a**) Apparent diffusion constants of PAmCherry-PBP1A molecules (TKL241) imaged on agarose pads containing β-lactam antibiotics (100 μg ml^−1^ each). The canonical targets for each antibiotic are indicated with filled red circles. (**b**) Apparent diffusion constants of PAmCherry-PBP1A molecules imaged in CS612, a strain containing multiple deletions for PBPs (CS109 Δ*dacB*/*pbp4* Δ*dacA*/*pbp5* Δ*dacC*/*pbp6* Δ*pbpG*/*pbp7* Δ*ampC* Δ*ampH*) on agarose pads containing various antibiotics. (**c**) Apparent diffusion constants of PAmCherry-PBP1A molecules imaged in the wild-type strain background CS109. Error bars indicate the s.d. of 1,000 bootstrap samples.

To determine whether PBP1A dynamics depend on the protein's catalytic activity, we tracked PBP1A motion on treatment with cefsulodin, a β-lactam antibiotic that inhibits the transpeptidase activity of PBP1A/B, at a concentration four times the minimum inhibitory concentration (Fig. 2a). We observed a significant decrease in diffusive motion in both the presence (Fig. 2a, *P*<0.0001, permutation test) and absence (Supplementary Fig. 5, *P*<0.0001, permutation test) of PBP1B, suggesting that the motion of PBP1A reflects its enzymatic state inside living cells and that motion slows when PBP1A's transpeptidase activity is inhibited.

Since other PBPs have been shown to interact with PBP1A^11^, we sought to determine whether inhibiting other PBPs would affect PBP1A dynamics. PBP2 interacts with PBP1A *in vitro*^11^, but the PBP2-specific inhibitor mecillinam had no effect on PBP1A diffusion even at concentrations three orders of magnitude above the minimum inhibitory concentration (Fig. 2a). Simultaneous treatment with both cefsulodin and mecillinam quantitatively mimicked the reduction in motion under cefsulodin alone (Fig. 2a). Interestingly, ampicillin, a broad-spectrum β-lactam that binds all PBPs, did not elicit a decrease in PBP1A motion at concentrations ∼30 times that shown to bind PBP1A^8^ (Fig. 2a), suggesting that inhibition of other PBPs beyond PBP1A/B restores the diffusivity of inhibited PBP1A. To test this hypothesis, we treated the cells with cefmetazole, which inhibits all PBPs except PBP2 (ref. 28). In agreement with our hypothesis, PBP1A molecules in these cells behaved similarly to molecules in cells exposed to ampicillin rather than cefsulodin (Fig. 2a). Similarly, cefmetazole+mecillinam treatment did not significantly affect PBP1A motion (Fig. 2a, *P*=0.085, permutation test). However, cefsulodin+ampicillin treatment resulted in a reduction in diffusion similar to that evoked by cefsulodin alone (Fig. 2a), which may be due to the fact that PBP1A is more sensitive to cefsulodin than to ampicillin^29^ or is able to overcome the effect of inhibiting other PBPs. Furthermore, cefsulodin+cefmetazole treatment resulted in a diffusion constant intermediate between those of the cefsulodin and the untreated/ampicillin cases (Fig. 2a). While the effects of cefmetazole on the rest of the PBPs besides PBP2 may not quantitatively match those of ampicillin, the relief of some of the cefsulodin-mediated inhibition of diffusion in combination with cefmetazole hints that PBP2 may also be partially responsible for the slowing of PBP1A diffusion. Taken together, these data suggest that PBP1A motion is affected by the activity of at least one other PBP.

### Other PBPs modify the effect of antibiotics on PBP1A

To test whether another PBP interacts with PBP1A and thereby affects its motion, we measured PBP1A dynamics in a previously characterized strain (CS612)^7^ that lacks many of the known low molecular weight PBPs (PBP4, 5, 6, 7, AmpC and AmpH). In this strain, treatment with cefsulodin, ampicillin, or cefsulodin+ampicillin did not affect PBP1A diffusion (Fig. 2b). In comparison, the CS612 background strain (CS109), which contains the full complement of PBPs (Fig. 2c), displayed a response to these antibiotics that mirrored those of *E. coli* MG1655 (Fig. 2a). The distinct behaviour of PBP1A under cefsulodin treatment in CS612 cells raises the possibility of direct interactions between PBP1A and one or more of the low molecular weight PBPs in wild-type cells, although there could be indirect effects on PBP1A in CS612 cells, for instance through changes in cell wall structure. Regardless, the presence of one of the affected PBPs is required for the observed reduction in PBP1A diffusion under cefsulodin treatment.

### LpoA regulates PBP1A dynamics

**Figure 3:**
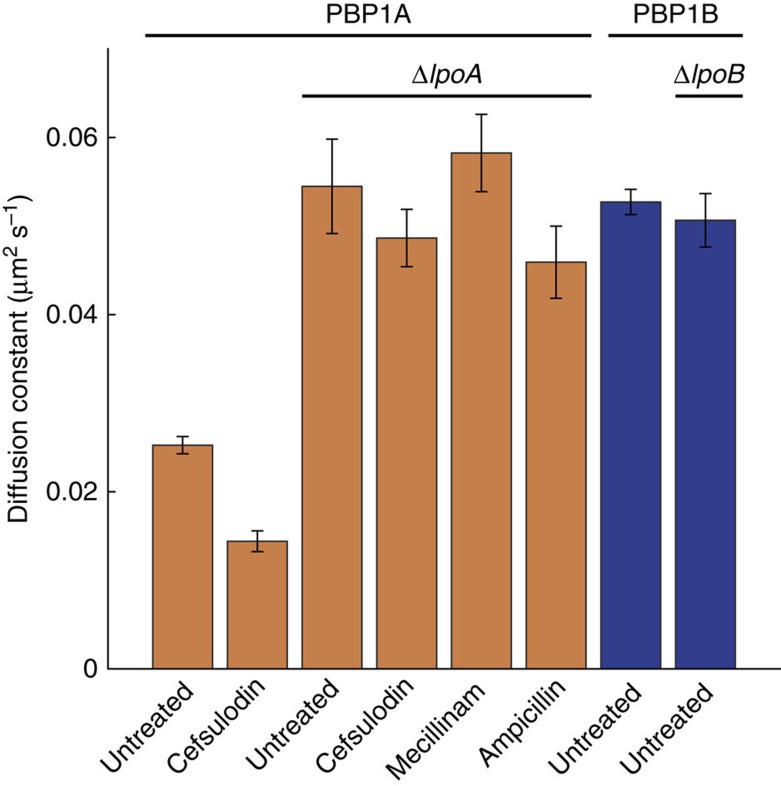
PBP1A dynamics and activity are regulated by LpoA *in vivo*. Apparent diffusion constants of PAmCherry-PBP1A molecules (orange) imaged in a strain deleted for *lpoA* (TKL242) and treated with cefsulodin, mecillinam, or ampicillin (100 μg ml^−1^ each). Diffusion constants of PAmCherry-PBP1B molecules (blue) were determined with and without *lpoB* (strain TKL243). Error bars indicate the s.d. of 1,000 bootstrap samples.

The outer membrane lipoprotein LpoA is essential for PBP1A function^13^,^14^, directly interacts with PBP1A, and stimulates the two catalytic activities of PBP1A *in vitro*^15^. To test whether the LpoA-mediated activation of PBP1A causes a change in PBP1A dynamics *in vivo*, we deleted *lpoA* and performed sptPALM on PAmCherry-PBP1A (Fig. 3). Strikingly, the rate of PBP1A diffusion (Fig. 3) increased to a level quantitatively similar to that of PBP1B (Fig. 1c). This increase in motion was not significantly affected by treatment with cefsulodin, mecillinam, or ampicillin (Fig. 3, *P*>0.2, permutation test). These data suggest that PBP1A dynamics are regulated by LpoA *in vivo*, and either that PBP1A slows to associate with LpoA or that LpoA is required to activate PBP1A enzymatic activity (thereby slowing the motion of PBP1A); our cefsulodin experiments (Fig. 2a) support the former possibility, since blocking PBP1A transpeptidase activity slows PBP1A.

### PBP1B is unaffected by perturbations during cell elongation

In contrast to our observations of PBP1A (Fig. 2), treatment with antibiotics did not appreciably affect the dynamics of PBP1B (Supplementary Fig. 6). Furthermore, deletion of PBP1B's lipoprotein regulator *lpoB* did not change PBP1B diffusion (Fig. 3). The quantitative similarity between the diffusion constant of PBP1B (with or without LpoB) and the fast diffusion constant of PBP1A without LpoA (Fig. 3) is consistent with the interpretation that PBP1B is less active during elongation, although it is also possible that PBP1B diffusion is simply not modulated in the same manner as PBP1A.

### PBP1A step sizes separate into two diffusive states

**Figure 4:**
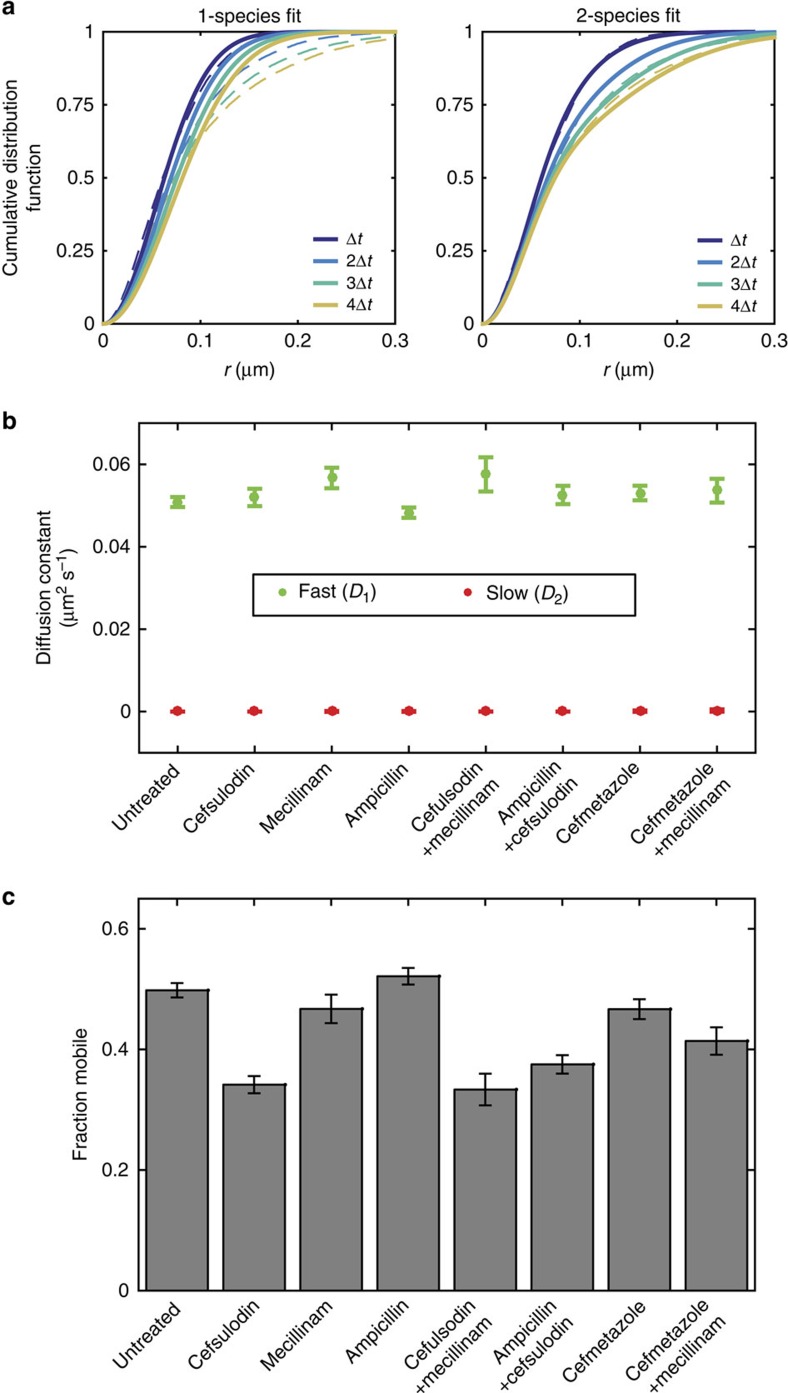
Relative PBP1A mobility can be explained by a shift in the mobile fraction. (**a**) The cumulative distribution function (dotted lines) of step sizes (*r*) for PBP1A molecules (*n*=3,177 molecules, TKL241) was calculated for Δ*t* of 1, 2, 3, or 4 frames (30, 60, 90, or 120 ms, respectively). These four distributions were simultaneously fit to models with a single species (left) or with two diffusing species (right). Model equations are given in the Methods. (**b**) The fits with two diffusing species resulted in a fast diffusion constant and a slow diffusion constant that was indistinguishable from localization error. (**c**) The fraction of mobile molecules (governed by the fast diffusion constant, *D*_1_) mimics the effective diffusion constant fitted from the MSD. In **b** and **c**, error bars indicate the s.d. of 1000 bootstrap samples.

We observed that the MSD of PBP1A (Fig. 1b) saturates at a level lower than would be expected due to the geometric constraints of the cell (Supplementary Fig. 7). We hypothesized that some PBP1A molecules move at a much slower rate than others, reducing the MSD over time; these slow-moving molecules would be enriched in the population of long TIRF tracks because faster-moving molecules are more likely to move out of the imaging field, terminating their tracks. Monte Carlo simulations based on this model of molecular switching between a fast state and a slow state produced MSD traces of molecules observable within the TIRF field that agreed with our experimental measurements (Supplementary Fig. 7). Since single-molecule tracks are often too short or not localized well enough to perform detailed analysis on individual molecules^30^, we analysed the bulk properties of our experimental data through the step-size cumulative distribution function. We fit the observed cumulative distribution function for 30-, 60-, 90-, and 120-ms intervals using either a model with one molecular species governed by one diffusion constant or a model with two molecular species with different diffusion constants (Fig. 4a, Online Methods). Our PBP1A measurements were much better fit by a model with two molecular species, one with a fast diffusion constant of ∼0.05 μm^2^ s^−1^ and one with a much lower diffusion constant indistinguishable from localization error (∼30 nm; Supplementary Table 1). Interestingly, when the step-size cumulative distribution functions for PBP1A under varying antibiotic treatments were fit in the same way, the estimates for localization error, slow diffusion, and fast diffusion converged to similar values (Fig. 4b, Supplementary Table 1). The only difference was in the estimate for *α*, the fraction of molecules undergoing fast diffusion (Fig. 4c), which mirrored the overall diffusion constant under antibiotic treatments (Fig. 2a). As an alternative probe of the number of diffusive states, we carried out a variational Bayes single-particle tracking analysis^31^, which also suggested that the optimal model contains two diffusive states of PBP1A (Supplementary Table 2, Supplementary Fig. 8). Altogether, these results suggest that perturbations to PBP1A diffusivity affect the proportion of molecules that undergo fast diffusion as opposed to remaining static.

## Discussion

Using *in vivo* single-molecule tracking, we observed a link between the catalytic activity and spatial dynamics of PBP1A during the elongation process of cell growth. However, PBP1A and PBP1B inactivation with cefsulodin had no effect on growth rate during elongation (Supplementary Fig. 9), in agreement with the previous observation that MreB speed is not affected by cefsulodin treatment^22^, suggesting that either PBP1A is not essential during elongation or that another peptidoglycan synthase is active in its place. This inference agrees with the previous observation that PBP1A inactivation has no effect on MreB dynamics^22^, a proxy for cell wall growth rate^21^,^32^. PBP1B is thought to be more important than PBP1A for cell division. To address whether the process of cell division affected our measurements, we re-analysed PBP1A/1B behaviour separately for the subpopulations of cells with length below and above the median length of the entire population. While the diffusion constant was unaffected for PBP1A, there was some evidence for a decrease in PBP1B mobility in longer cells (Supplementary Fig. 10, *P*=0.05, permutation test). Together with our observations of the absence of changes in the dynamics of PBP1B molecules under antibiotic treatment (Supplementary Fig. 3) and loss of LpoB (Fig. 3), these data suggest that PBP1B's activity in elongation is minimal, although further evidence is required. Note that imaging of PBP1B behaviour during cell constriction is challenging, because molecules that are more than ∼100–150 nm from the cover slip cannot be observed using TIRF microscopy.

In the absence of the other PBP1 protein, the terminal phenotypes of PBP1A and PBP1B inactivation are similar, with membrane bulging and eventual lysis at the poles or nascent septa near midcell^14^. Since the minimum inhibitory concentration of cefsulodin for Δ*mrcB* is much lower than for Δ*mrcA*^14^, it is likely that PBP1A can only partially complement the essential activity of PBP1B. This partial complementation is also evident in the elevated frequency of lysis^33^ and reduced mechanical stability of a Δ*mrcB* mutant^34^. Altogether, these data imply that the redundant function of PBP1A is to compensate for the loss of PBP1B activity during division, and that neither PBP1A nor PBP1B is essential during elongation. Immunofluorescence imaging of PBP1A and PBP1B supports this interpretation, as PBP1B is more strongly localized to division sites^12^, and the septal localization of PBP1A is enhanced when PBP1B is absent^11^.

In this study, measurements of PBP1A dynamics demonstrate that the diffusive state of a protein can be indicative of its enzymatic state *in vivo*, providing an indirect assay of protein function and regulation, which are typically not accessible in living cells. While the relationship between enzymatic state and molecular motion was not simple in the present study, our results highlight the utility of imaging for dissecting the complex relationships among all components of the cell wall synthesis machinery. Our previous observations^23^ of the contrast between the slow, directed motion of MreB and the fast, diffusive motion of PBP2 imply that the coordination of cell wall synthesis need not require the colocalization of individual components of this process. The current study of PBP1A dynamics suggests that other components likely exhibit similar diffusive behaviour and that short, transient associations are likely common to the coordination of this multi-enzyme process. For essential processes, dynamic associations buffer the cell against changes in molecular concentrations and perturbations with antibiotics, and loosen requirements on the stoichiometry of complexes. We propose that this mechanism is general to many macromolecular complexes and is especially beneficial when the components are present at low abundances.

Our study further emphasises that the coordination of cell wall synthesis involves multiple layers of regulation and interactions between components. For example, the varied dynamics of PBP1A indicate that several *in vivo* behaviours exist: (1) fast diffusion in the absence of LpoA regulation, (2) a mixture of behaviours under normal growth conditions (elongation), and (3) slow diffusion with nearly stationary molecules under cefsulodin inhibition. The drastic change in PBP1A mobility in Δ*lpoA* cells (Fig. 3) supports the role of outer-membrane regulation in cell wall synthesis, and it remains to be determined whether this effect is a direct result of the physical interaction between LpoA and PBP1A or a change in PBP1A activity. In addition, our results suggest the existence of a complex network of interactions among the PBPs, as evidenced by the effect of the low molecular weight PBPs on PBP1A behaviour under cefsulodin treatment (Fig. 2b). This effect could be direct, whereby the other PBPs interact with cefsulodin-bound PBP1A to extend its time in a static state, or indirect through modifications to cell wall ultrastructure or PBP1A conformation. Our data suggest that this static state involves activation by LpoA, PBP1A binding to its substrate (or to the analogue cefsulodin), and the presence of one or more low molecular weight PBPs, whose specific roles in cell wall synthesis have been challenging to uncover due to redundancy; sptPALM will likely continue to prove a powerful means of addressing this challenge.

The mechanistic link between cell wall synthesis and processive MreB motion, which was recently shown to depend on RodZ^35^, remains to be fully elucidated but has important implications for cell growth and shape determination. A motor-like enzymatic cycle similar to eukaryotic actinomyosin systems may not be required; localization of MreB to areas of negative membrane curvature could explain circumferential motion^21^. Alternatively, an ordered set of peptidoglycan-synthesis steps may underlie the processive nature of MreB motion, as hinted at by our observations of high molecular weight PBP interactions with low molecular weight PBPs (Fig. 2b). Uncovering the couplings among the different enzymatic activities is likely to be critical for understanding the mechanisms of action of current antibiotics and how best to select new molecules that disrupt cell wall assembly^36^. Our results indicate that *in vivo* single-molecule tracking is a promising method for characterizing the cytoskeletal–peptidoglycan interaction network and can be broadly applied to study dynamic processes involving the coordination of many proteins.

## Methods

### Strain construction

The strains and plasmids used in this study are described in Supplementary Tables 3 and 4, respectively. All of the strains used in imaging experiments expressed the relevant fluorescent protein from the native locus, fully replacing the wild-type protein. Plasmids were constructed using enzymatic assembly methods^37^. Expression plasmids were constructed using a low-copy plasmid with a pSC101 origin (pRM102)^38^, and coding sequences for the relevant genes were amplified from *E. coli* MG1655 with the appropriate homology regions for assembly. Fluorescent protein fusions were introduced into the chromosome using allelic exchange methods with suicide plasmids^39^ or through Lambda Red recombination^40^. For allele exchange, the desired sequences for integration were amplified via PCR and cloned into pDS132 (ref. 39). MFD*pir*^41^ cells were transformed with the resulting plasmids and used for conjugative transfer into the recipient strain. The resulting merodiploids were selected on lysogeny broth plates supplemented with 100 μg ml^−1^ chloramphenicol. Strains that had lost the integrated plasmid (and *sacB*) through homologous recombination were selected on lysogeny broth plates containing 5% sucrose. All chromosomal modifications were confirmed by amplification and sequencing of the targeted region. Gene deletions were introduced through P1 transduction using lysates made from strains in the KEIO nonessential deletion library^42^.

To construct the PAmCherry-PBP1A fusion, we optimized an eight-amino acid linker between PAmCherry and PBP1A via PCR using oligonucleotides with degenerate bases. Briefly, we amplified a PCR fragment that includes Δ*yrfD*::kan (a neutral disruption directly upstream of *mrcA* to be used for selection), the promoter of *mrcA*, and the PAmCherry sequence using a reverse primer of the following form: (PAmCherry-END)-VNNVNNVNNVNNVNNVNNVNNVNN-(PBP1A-BEGIN). To select for linkers resulting in functional fusions, the resulting fragment was used to replace the native copy of *mrcA* in a Δ*mrcB* background (TKL238) through Lambda Red recombination. The resulting clones were screened for maximal fluorescence; the linker used for all subsequent experiments had the amino-acid sequence RGNQHPQ. Construction details for each strain are provided in the Supplementary Methods.

### Microscopy

Imaging was performed on a total internal reflection fluorescence (TIRF) microscope built with a Ti-E Eclipse stand (Nikon Instruments, Inc., Melville, NY, USA). A Plan Apo Lambda 100X DM (NA 1.45) (Nikon) objective was used to acquire phase-contrast images concurrently with TIRF images. CUBE diode 405-nm and Sapphire OPSL 561-nm lasers (Coherent, Santa Clara, CA, USA) were combined into an optical fibre and into a TIRF illuminator (Nikon) attached to the microscope stand. Shuttering of laser illumination was controlled with an acousto-optic tuneable filter (AA Optoelectronics, Orsay, France) before the fibre coupler. Images were acquired with an iXon3+ 887 EMCCD (Andor Technology, South Windsor, CT, USA) camera, and synchronization between components was achieved using μManager 1.4 (ref. 43) with a microcontroller (Arduino, Almuñécar, Spain).

### Single-particle imaging

Cells expressing fluorescently labelled proteins were grown to saturation overnight in the rich medium EZ-RDM (Teknova, Hollister, CA, USA)^44^ with 0.2% glucose and then diluted 1:100 in fresh medium and incubated with shaking at 30 °C for 3.5 h. Cells were spotted onto 1% agarose pads with EZ-RDM+0.2% glucose and covered with argon plasma-cleaned coverslips. For drift correction, phase-contrast images were taken interlaced with fluorescence images. To capture PBP1A/1B/2 dynamics, image acquisition was alternated between: 1) an exposure with 561-nm laser at ∼1 kW cm^−2^ to image single PAmCherry molecules and 2) a simultaneous exposure with a brightfield light-emitting diode and 405-nm laser (∼0.50 kW cm^−2^ for PBP1A and PBP2, ∼0.05 kW cm^−2^ for PBP1B) to capture phase-contrast images and to photoactivate PAmCherry molecules. In single-particle tracking experiments under antibiotic treatment, agarose pads were cast with the appropriate antibiotic (cefsulodin, mecillinam, ampicillin, and cefmetazole at 100 μg ml^−1^) and cells were directly spotted from liquid culture.

### Single-particle tracking analysis

Images were analysed computationally to generate single-particle tracks using the u-track package^45^ in MATLAB (MathWorks, Natick, MA, USA). Gaussian mixture-model fitting was used for particle detection and the routines were modified for multicore processors. Drift between images was corrected using the cross-correlation on the phase-contrast images after smoothing using the ‘spaps' MATLAB function. Tracks were calculated using the ‘costMatLinearMotion' cost function in u-track. Only tracks detected within cellular boundaries (determined from segmentation of phase-contrast images) were used for analysis. For PBP1A/B molecules, tracks persisting for 4–12 time points were considered for further analysis. MSD was calculated from positions in the plane; the effects of the curvature of the cell within the TIRF field represent only a small correction (Supplementary Fig. 7). An estimate for the diffusion constant was determined from a linear fit to the first four points of the MSD. Errors in diffusion constant calculation were estimated through 1000 bootstrap samples of single-molecule tracks. Statistical significance between conditions or treatments was calculated using a permutation test with bootstrap sampling.

### Step-size cumulative distribution function fitting

Molecular tracks from a given condition were aggregated and an empirical cumulative distribution function was calculated for stepsizes (function ‘ecdf' in MATLAB) with time steps of 30, 60, 90, and 120 ms. The resulting set of cumulative distribution functions was fit to a single function using the ‘NonLinearLeastSquares' method in MATLAB to a model with one or two diffusing species as given by:


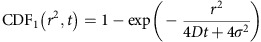



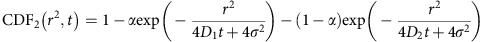


where *r*^2^ is the squared displacement, *t* is the time step, *D* is the diffusion constant, *σ* is the localization error, and α is the fraction of molecules diffusing with constant *D*_1_ (fast species).

### Single-cell imaging of growth

Exponentially growing cells in EZ-RDM+0.2% glucose at 30 °C were loaded into a B04a bacterial microfluidic chip in a CellASIC ONIX system (EMD Millipore, Hayward, CA, USA). Cells were grown in EZ-RDM+0.2% glucose for 10 min at 30 °C while being imaged in phase-contrast every 30 s. Then, the medium was switched to EZ-RDM+0.2% glucose with 100 μg ml^−1^ cefsulodin and imaging continued for another 50 min. Cell contours were automatically extracted from phase-contrast images using a custom MATLAB package. Cell length over time, *L*(*t*), was smoothed with a moving average window of 10 time points and instantaneous growth rate was calculated as 1/*L dL*/*dt*.

### Data availability

The data that support the findings of this study are available from the corresponding author on reasonable request.

### Code availability

All code is available on request from the authors.

## Additional information

**How to cite this article**: Lee, T. K. *et al*. Single-molecule imaging reveals modulation of cell wall synthesis dynamics in live bacterial cells. *Nat. Commun.* **7**, 13170 doi: 10.1038/ncomms13170 (2016).

## Supplementary Material

Supplementary Information Supplementary Figures 1-10, Supplementary Tables 1-4, Supplementary Methods, Supplementary References.
