# Supplementary material for: Single-molecule imaging reveals modulation of cell wall synthesis dynamics in live bacterial cells
Source: Nat Commun. 2016 Oct 24;7:13170. doi: 10.1038/ncomms13170 (PMC5078992; doi:10.1038/ncomms13170)
Supplement: Supplementary Information — Supplementary Figures 1-10, Supplementary Tables 1-4, Supplementary Methods, Supplementary References. [file ncomms13170-s1.pdf]

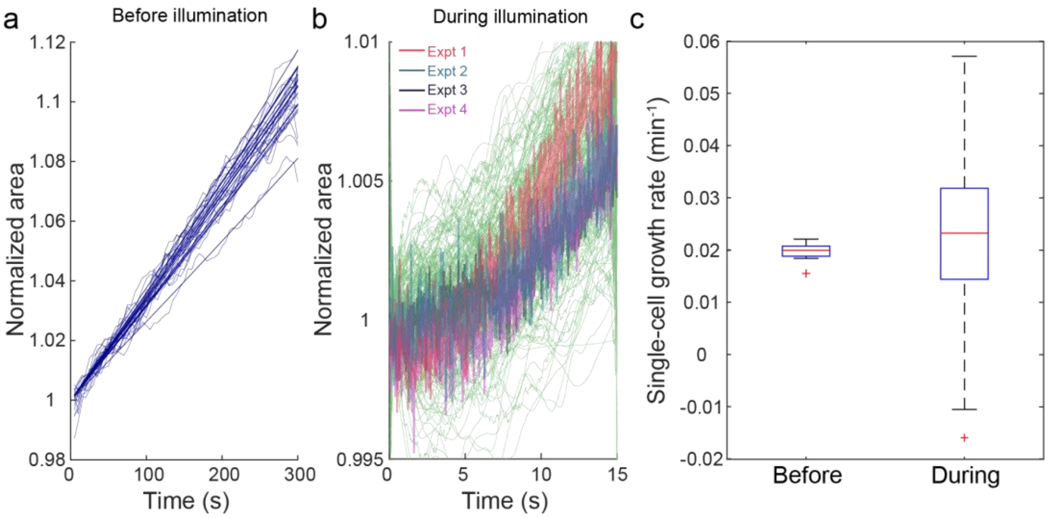

Supplementary Figure 1 Single-cell elongation rate, as assayed during sptPALM imaging. Mean elongation rate across single cells was evaluated according to an exponential fit to the projected cell area increase over time throughout a 5-min interval directly before and during a 15-s interval of illumination with the 405-nm activation laser.

a) Area traces were smoothed over 30-s intervals (10 frames) prior to fitting (straight lines). Plotted is the area of each cell normalized to the fitted value of the initial area. The mean doubling time is 35 min (*n* = 17 cells).

b) Area traces (thin green curves) were smoothed over 0.3-s intervals (20 frames) prior to fitting. Thick curves are the average area traces (unsmoothed) for the four experiments used to compute the diffusion constant for PBP1A in Fig. 1C (*n* = 57-65 cells in each experiment). Plotted is the area of each cell normalized to the fitted value of the initial area. The doubling time estimated from the four average area traces was 37.5±9.5 min.

c) Box plot of elongation rates for cells in (A) and (B). The elongation rate during illumination was more noisy due to the short imaging time, but mean values were similar.

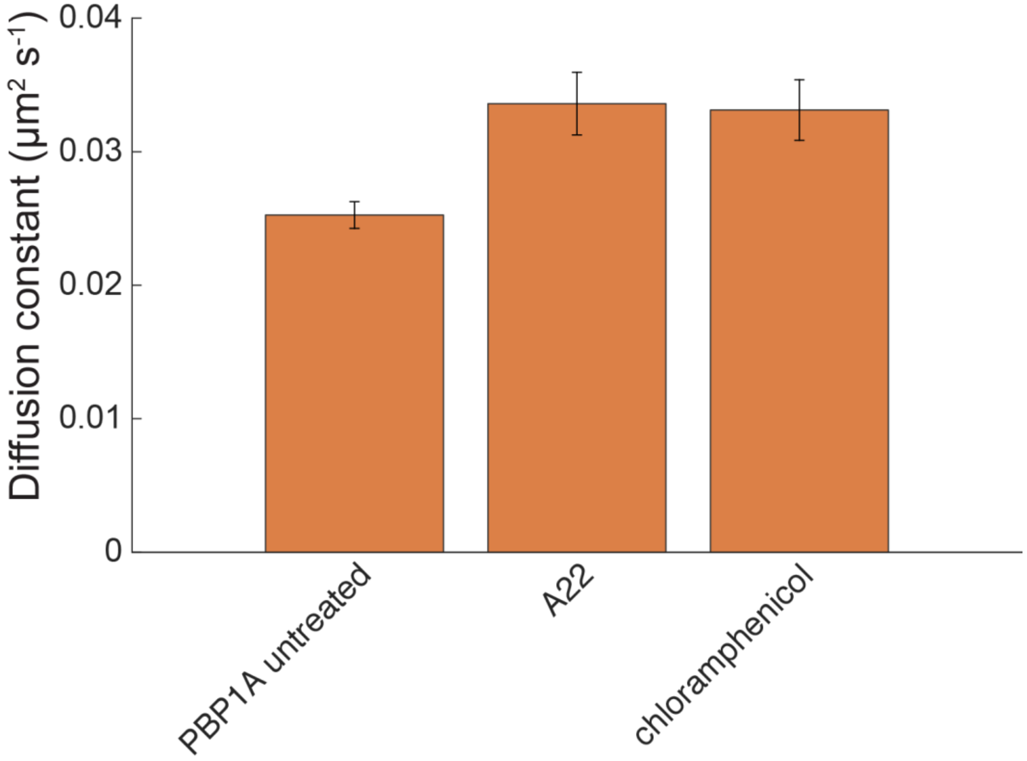

Supplementary Figure 2 Apparent diffusion constants of PAmCherry-PBP1A molecules imaged in TKL130 cells treated with the antibiotics A22 (100 µg/ml) or chloramphenicol (100 µg ml^{-1}). Error bars indicate the standard deviation of 1000 bootstrap samples.

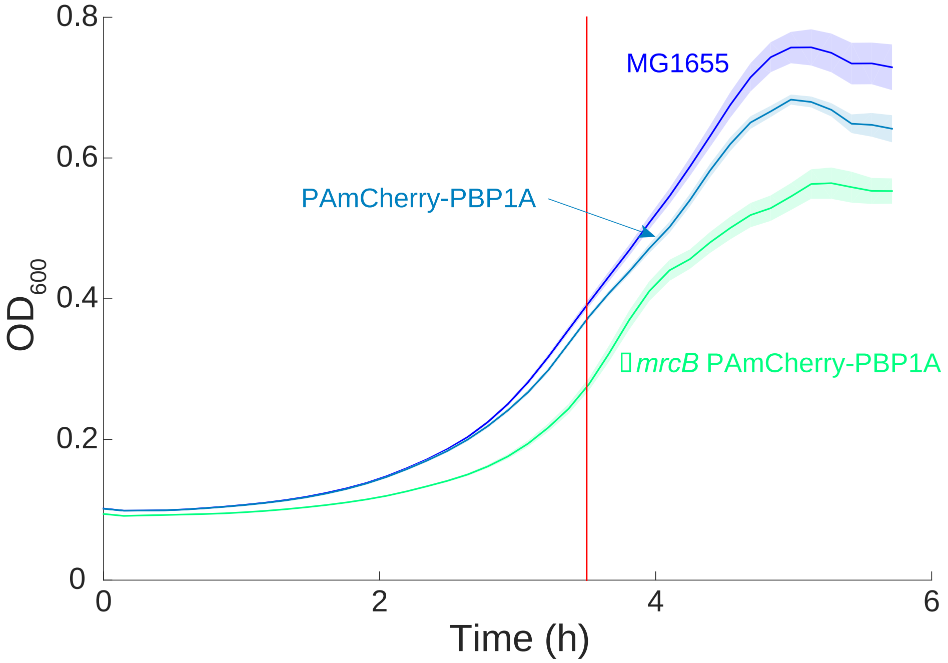

Supplementary Figure 3 Growth curves of MG1655, TK241 (PAmCherry-PBP1A, Table S3), and TK240 (Δ*mrcB* PAmCherry-PBP1A, Table S3) cells measured in a plate reader. Curves are means (*n* = 3) with the shaded areas representing one standard deviation above and below the mean. Vertical red line indicates the time point of sptPALM imaging.

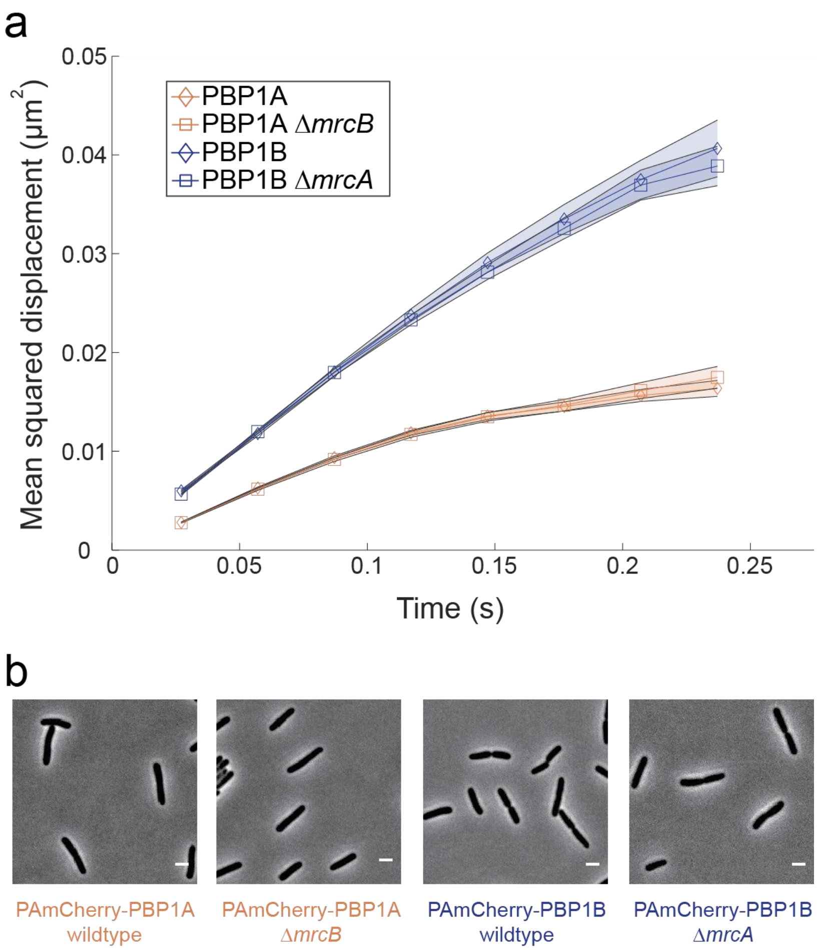

Supplementary Figure 4 PBP1A and PBP1B dynamics do not depend on the presence of the other protein. (**a**) Mean squared displacement of PAmCherry-PBP1A in wild-type (*n* = 3177 molecules, TKL241) and Δ*mrcB* (lacking PBP1b; *n* = 1994 molecules, TKL240) backgrounds and PAmCherry-PBP1B in wild-type (*n* = 898 molecules, TKL211) and Δ*mrcA* (lacking PBP1a; *n* = 1491 molecules, TKL239) backgrounds. These data are also represented in Figure 1c of the main text. Shaded areas represent the standard error of the mean for a given time point. (**b**) Phase-contrast images of the strains used for single-molecule imaging. Scale bar: 2 μm.

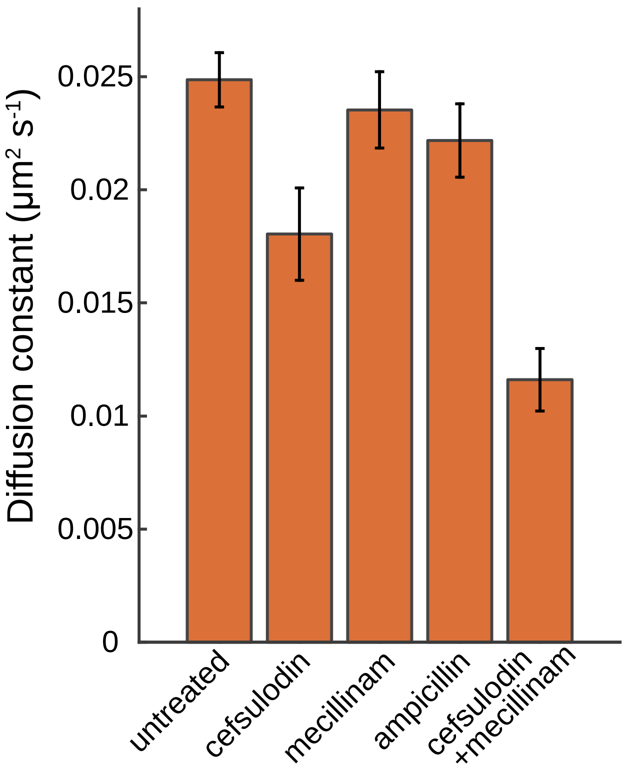

Supplementary Figure 5 The effect of antibiotics on PBP1A dynamics is not disrupted by the absence of PBP1B. Apparent diffusion constants of PAmCherry-PBP1A molecules in a Δ*mrcB* background (TKL240) imaged on agarose pads containing β-lactam antibiotics (100 μg ml^{-1} each). Error bars indicate the standard deviation of 1000 bootstrap samples.

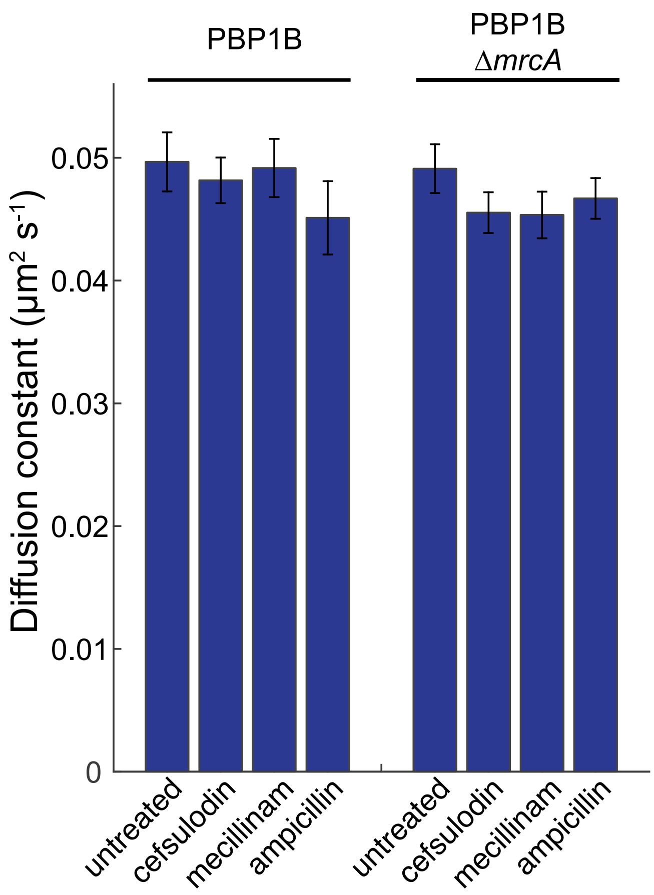

Supplementary Figure 6 PBP1B dynamics are not affected by antibiotics during cell elongation. Apparent diffusion constants of PAmCherry-PBP1B molecules (TKL211) or PAmCherry-PBP1A in a Δ*mrcA* background (TKL250); cells were imaged on agarose pads containing β-lactam antibiotics (100 μg ml^{-1} each). Error bars indicate the standard deviation of 1000 bootstrap samples.

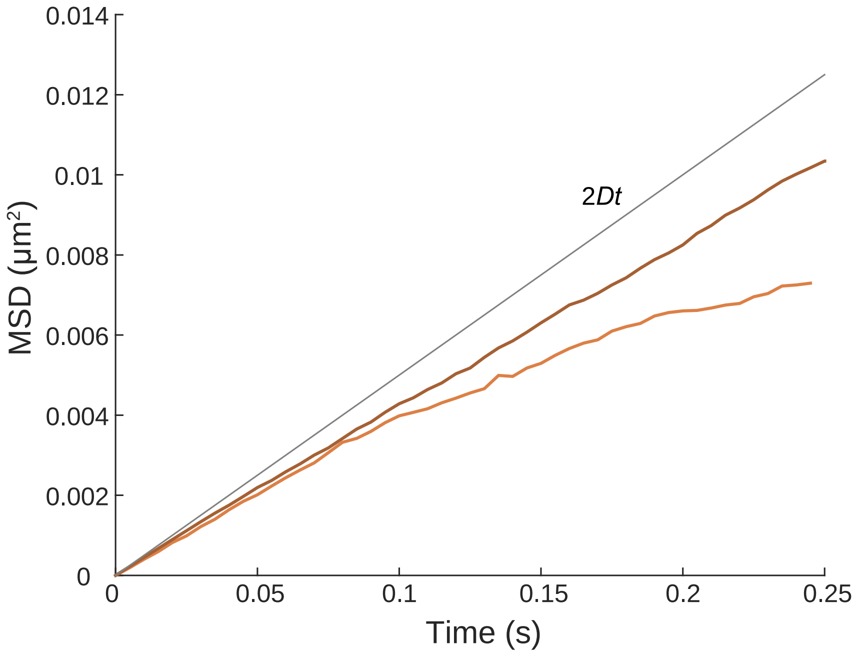

Supplementary Figure 7 Quantification of the effects of the curvature of the cell and of having a slow-moving subpopulation of molecules on MSD. To simplify interpretation, we consider only one-dimensional motion. Gray line shows the expected MSD for molecules with a diffusion constant D = 0.025 μm^2/s. The dark orange line shows the effects of cell curvature within the TIRF field. Shown is the MSD projected onto the plane from a Monte Carlo simulation of 20,000 molecules diffusing in the circumferential direction with D = 0.025 μm^2/s along a cylindrical surface in which only the duration of tracks that remained in the TIRF field (defined as 0.1 μm from the bottom of the cell) were used to compute the MSD. The projected TIRF MSD is expected to plateau at 0.032 μm^2. The bright orange line shows the projected MSD in a Monte Carlo simulation in which molecules switched randomly between a fast-diffusion state with D = 0.05 μm^2/s and a static state with D = 0 μm^2/s, representing a fast-moving fraction of α = 0.5. The MSD starts to plateau because slow-moving molecules are enriched in the population of long TIRF tracks;

fast-moving molecules are more likely to move out of the TIRF field, thereby terminating their tracks. Monte Carlo simulations were performed using a time step of dt = 5 ms and a grid spacing of 31 nm.

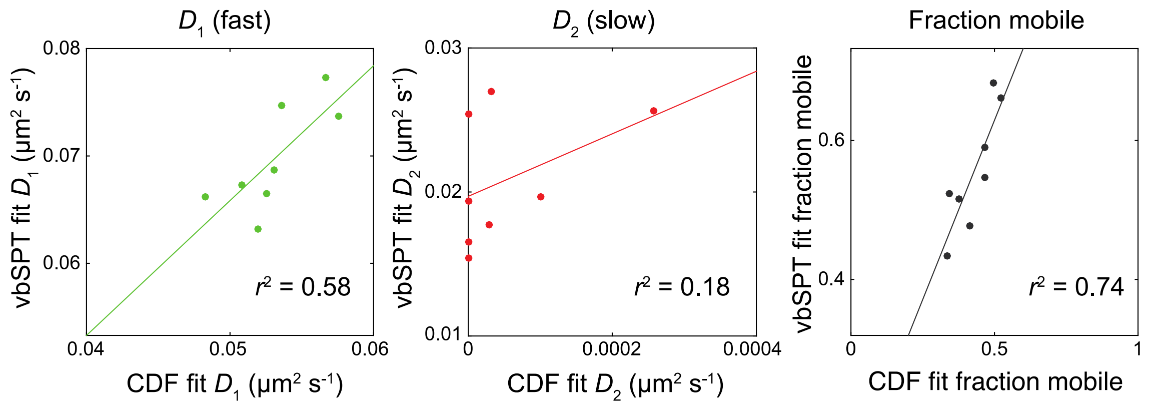

Supplementary Figure 8 Comparison of diffusion constant estimates and fractions of mobile molecules from fitting cumulative distribution functions versus using the variational Bayes single-particle tracking (vbSPT) package. Each point represents a condition as presented in Tables S1 and S2, and each line is the best fit to the data. vbSPT returned the best global model as having two states for all conditions listed, where the maximum number of conditions considered was limited to five.

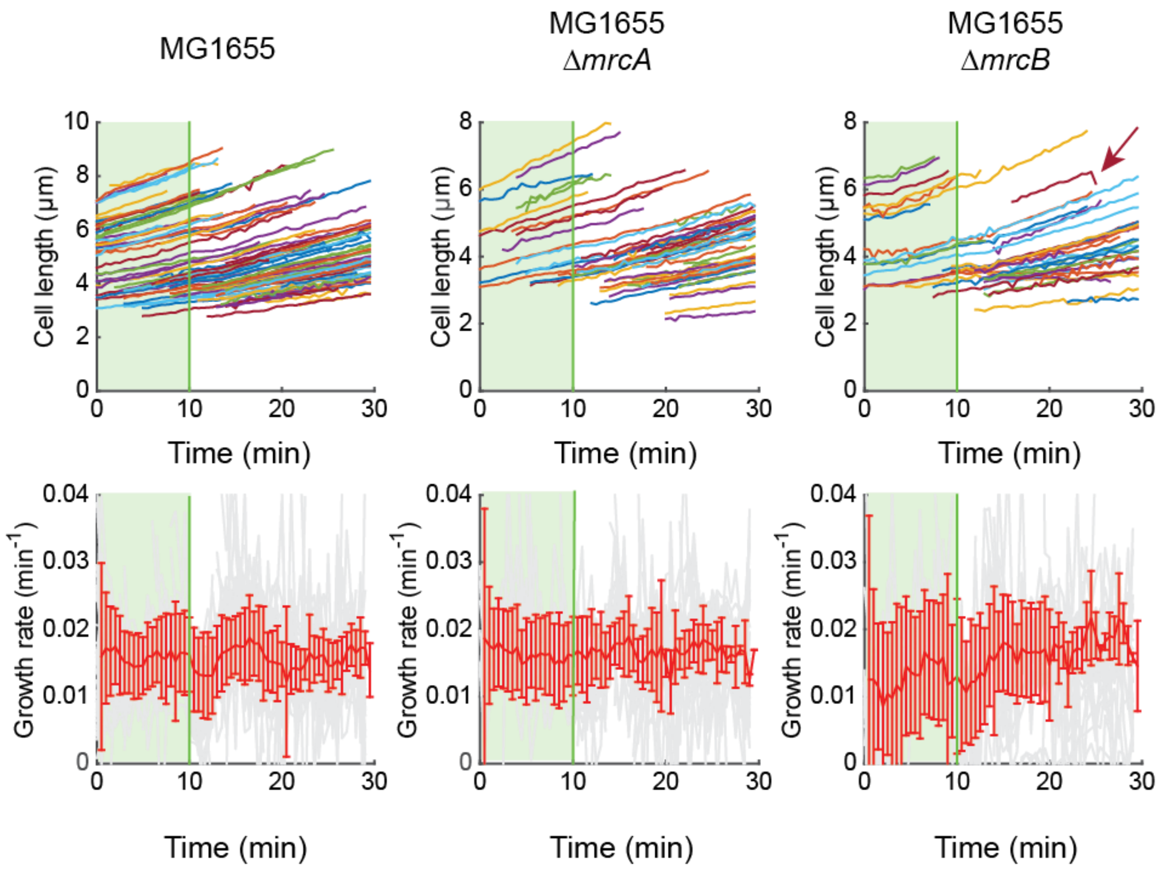

Supplementary Figure 9 *E. coli* growth rate during elongation is not affected by cefsulodin. *E. coli* cells were loaded into a CellASIC microfluidic device and growth was observed over 1 h in EZ-RDM + 0.2% glucose at 30 ˚C. From t = 0 to 10 min, cells elongated at a constant rate in medium without cefsulodin. At 10 min (green vertical line), the flow of medium was switched to include 100 µg ml^{-1} cefsulodin. Exponential growth rate (bottom) was calculated from the change in cell length (top) for single cells. Cells whose tracking initiates after t = 0 are predominantly daughter cells that appear due to a division event, with occasional cells coming into the imaging frame due to flow.

The Δ*mrcB* cell (red arrow) that shows a marked decrease in length in the final frame tracked was verified to be in the process of lysing. Single grey lines indicate

growth rates for individual cells, red lines indicate the average ($n \geq 3$) growth at each time point, and error bars represent standard deviation.

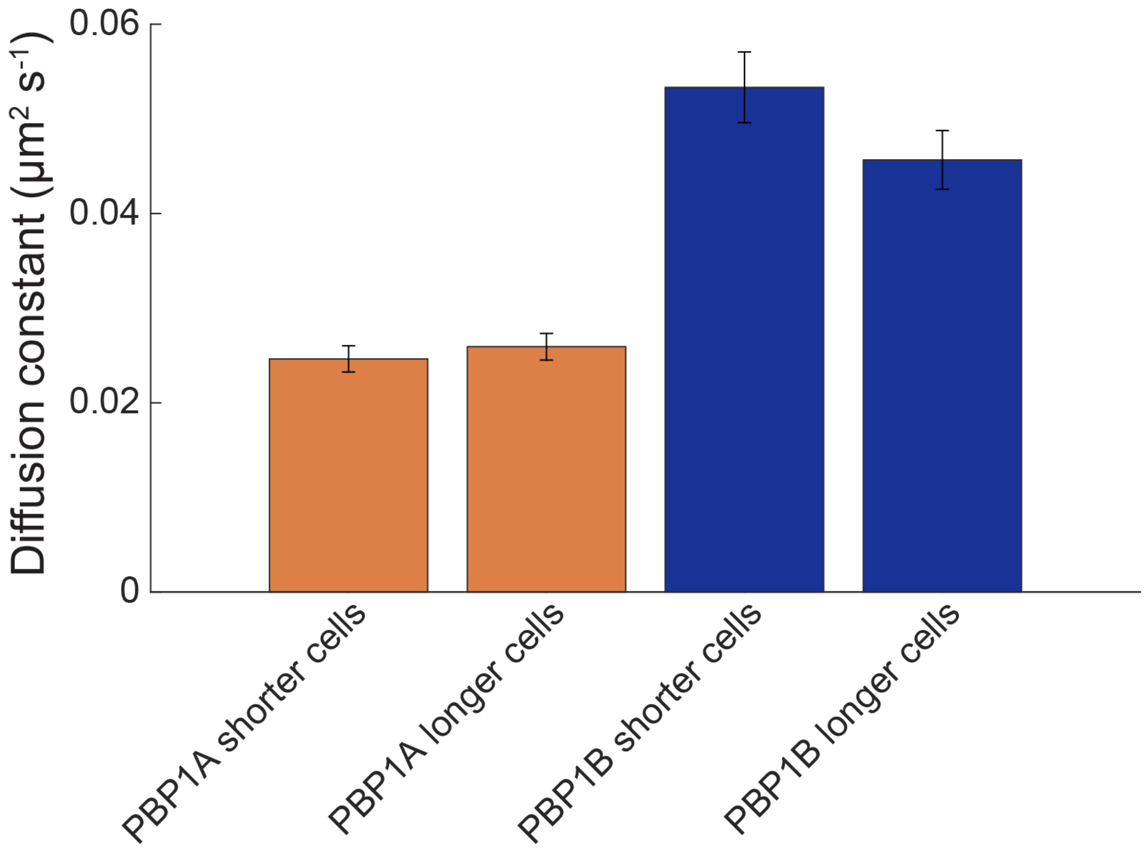

Supplementary Figure 10 The apparent diffusion constant for untreated cells in wild-type backgrounds (TKL241 for PBP1A and TKL211 for PBP1B) presented in Figure 1c was re-analysed by partitioning cells into groups above or below the median cell length (4.2 µm for TKL241 and 4.6 µm for TKL211). Error bars indicate the standard deviation of 1000 bootstrap samples. The difference between PBP1A cell lengths is not significant (p = 0.46), while there may be a significant difference in PBP1B mobility as a function of cell length (p = 0.05).

Supplementary Tables

Supplementary Table 1 Step-size cumulative probability distribution fitting results.

Condition	D_1 (μm^2 s^{-1})	D_2 (μm^2 s^{-1})	α (fraction)	σ (μm)
**untreated**	0.051 ± 0.0012	7.3e-12 ± 1.4e-11	0.50 ± 0.012	0.029 ± 0.00032
**cefsulodin**	0.052 ± 0.0021	3.6e-12 ± 7e-12	0.34 ± 0.014	0.025 ± 0.00037
**mecillinam**	0.057 ± 0.0025	3.2e-5 ± 1e-4	0.47 ± 0.024	0.029 ± 0.00054
**ampicillin**	0.048 ± 0.0013	2.9e-5 ± 8.7e-5	0.52 ± 0.014	0.026 ± 0.00039
**cefsulodin+mecillinam**	0.058 ± 0.0041	6.4e-7 ± 1.2e-5	0.33 ± 0.026	0.028 ± 0.00057
**ampicillin+cefsulodin**	0.053 ± 0.0022	1.4e-7 ± 3.5e-6	0.38 ± 0.015	0.024 ± 0.00040
**cefmetazole**	0.053 ± 0.0018	1e-4 ± 1.5e-5	0.47 ± 0.016	0.026 ± 0.00046
**cefmetazole+mecillinam**	0.054 ± 0.0029	2.6e-4 ± 2.7e-4	0.41 ± 0.023	0.027 ± 0.00065

Supplementary Table 2 Diffusion parameters estimated from vbSPT.

Condition	D_1 (μm² s⁻¹)	D_2 (μm² s⁻¹)	α (fraction)
**untreated**	0.0673	0.0194	0.683
**cefsulodin**	0.0632	0.0165	0.524
**mecillinam**	0.0773	0.027	0.547
**ampicillin**	0.0662	0.0177	0.661
**cefsulodin+mecillinam**	0.0737	0.0254	0.434
**ampicillin+cefsulodin**	0.0665	0.0154	0.515
**cefmetazole**	0.0687	0.0197	0.59
**cefmetazole+mecillinam**	0.0747	0.0256	0.477

Supplementary Table 3 Strains used in this study. Note that *yrfD* is nearby *mrcA* and hence was used to move the PAmCherry-PBP1A construct between strains using P1 transduction or Lamba-Red recombination.

**Strain**	**Description**	**Ref/source**
**MG1655**	*Escherichia coli* K-12 wildtype	CGSC #6300
**CS109**	W1485 *glnV rpoS rph*	[1]
**CS612-1**	CS109 Δ*dacB* Δ*dacA* Δ*dacC* Δ*pbpG* Δ*ampC* Δ*ampH*	[1]
**TKL130**	MG1655 *mrdA*::*PAmCherry-mrdA*	[2]
**TKL238**	MG1655 Δ*mrcB*::frt pKD46	This study, [3]
**TKL240**	MG1655 Δ*mrcB*::frt Δ*yrfD*::kan *PAmCherry-mrcA*	This study
**TKL241**	MG1655 Δ*yrfD*::frt *PAmCherry-mrcA*	This study
**TKL242**	MG1655 Δ*yrfD*::frt *PAmCherry-mrcA* Δ*lpoA*::kan	This study
**TKL258**	CS109 Δ*yrfD*::kan *PAmCherry-mrcA*	This study
**TKL259**	CS612 Δ*yrfD*::kan *PAmCherry-mrcA*	This study
**TKL211**	MG1655 *PAmCherry-mrcB*	This study
**TKL239**	MG1655 Δ*mrcA*::kan *PAmCherry-mrcB*	This study
**TKL243**	MG1655 *PAmCherry-mrcB* Δ*lpoB*::kan	This study

Supplementary Table 4 Plasmids used in this study.

Plasmid	Description	Ref/source
**pRM102**	pSC101 ori, cat, rrnB T1, MCS, tL3, CmR	[4]
**pDS132**	R6K ori, sacB, MCS, cat, CmR	[5]
**pJ401-PAmCherry**	PAmCherry codon optimised for *E. coli*, KanR	[2]
**pKD46**	repA101(ts) araBp-gam-bet-exo oriR101 bla	[3]
**pCP20**	ts-rep [cI857]lambda(ts) bla cat FLP	[3]
**pKC175**	pDS132 P_{mrcB}-*PAmCherry-mrcB*	This study
**pKC178**	pRM102 Δ*yrfD*::kan P_{mrcA}-*PAmCherry-mrcA*	This study

Supplementary Methods

Detailed Strain Construction

TKL238 (Table S3) is a Δ*mrcB* background used for Lambda-Red recombineering[3]. The Δ*mrcB*::kan allele was introduced into MG1655 through a P1 lysate made from the corresponding strain in the KEIO deletion library[6], and the kan cassette was cured using pCP20 (Table S4). The resulting strain was then transformed with the Lambda-Red helper plasmid pKD46.

pKC178 is a template for the PAmCherry-PBP1A fusion with a defined linker, GHGTGSTGSGSS, that was later used for linker optimization to ensure functionality of the fusion. Enzymatic assembly was performed with the four following components: 1) the pRM102 vector amplified with primers 5’-CAAGCTTGGCACTGGCCACG and 5’-AATGGCGATGACGCATCCTC, 2) the flanking regions of the neutral deletion Δ*yrfD*::kan from its corresponding KEIO deletion strain amplified with primers 5’-GCCTTTTTGCGTGGCCAGTGCCAAGCTTGTCAGTACGCCTTCCCAAGCGC and 5’-CACTGGAAATTTCCCATTTAGTTTCATTTG, 3) the codon-optimized PAmCherry sequence from TKL130 amplified with primers 5’-CTGCCCAAATGAAACTAAATGGGAAATTTCCAGTGGTAAGCAAGGGCGAAGAGGACAACA and 5’-TGCAACAGACTGCAAGGATCAAAAAATACTTTACGAACTTTGAGCTGCCGCTGCCGGTGC

, and 4) the *mrcA* sequence from MG1655 amplified with primers 5’-AAGTTCGTAAAGTATTTTTTGATCCTTG and 5’-CCCGGATATTATCGTGAGGATGCGTCATCGCCATTTTTATCAGGCTTATGGGGTTTCTGC.

TKL240 contains a chromosomally integrated PAmCherry-PBP1A construct with the optimized linker RGNQHPQN, which was generated through the following process. First, a PCR fragment was prepared containing Δ*yrfD*::kan, the *mrcA* promoter, and PAmCherry from pKC178 using primer 5’-CCAGGGCAAAAAATTAATTGGCGGGTTCATCAGTACGCCTTCCCAAGCGC and a primer with degenerate bases corresponding to possible linker sequences, 5’-CCCAGCAGAATGCAACAGACTGCAAGGATCAAAAAATACTTTACGAACTTNNBNNBNNBNNBNNBNNBNNBNNBCTTGTACAACTCATCCATGCCACC. This fragment was introduced via Lambda-Red recombination into TKL238 and selected for kanamycin resistance. The resultant clones were further selected via microscopy for clones expressing PAmCherry-PBP1A.

TKL241 was derived from TKL240 by curing the kan resistance cassette using pCP20.

TKL242 was derived from TKL241 by introducing P1 lysate prepared from the Δ*lpoA* strain in the KEIO deletion library.

TKL258 was created by transducing CS109 with P1 lysate prepared from TKL240.

TKL259 was created by transducing CS612-1 with P1 lysate prepared from TKL240.

pKC175 was created via enzymatic assembly using the following PCR fragments: 1) the pDS132 vector using primers 5'-AGCTCTCCCGGGAATTCCAC and 5'-AGCTCTCCCGGGAATTCCAC, 2) the *mrcB* promoter from MG1655 using 5'-GGCAGGTATATGTGATGGGTTAAAAAGGATCGATCCTTTGGTTTTCTCCCTCTCCCTGTG and 5'-CATCGGTTCTTCATCCTCATAGTCATCATA, 3) PAmCherry using 5'-ACGATTATGATGACTATGAGGATGAAGAACCGATGGTAAGCAAGGGCGAAGAGGACAACA and 5'-GAGGCTTACGCCCTTTGCCTTTGCCCTTACCTTTGCGCGGTGAGCTGCCGCTGCCGGTGC, and 4) the *mrcB* coding sequence from MG1655 using 5'-CCGCGCAAAGGTAAGGGCAAAGG and 5'-CATCGGTTCTTCATCCTCATAGTCATCATAATCGTCGT.

TKL211 was created through allele exchange in MG1655 using the suicide plasmid pKC175.

TKL239 was created through transduction of TKL211 using P1 lysate prepared from the Δ*mrcA*::kan strain of the KEIO deletion library.

TKL243 was created through transduction of TKL211 using P1 lysate prepared from the Δ*lpoA*::kan strain of the KEIO deletion library.

Supplementary References

1. Denome, S.A., Elf, P.K., Henderson, T.A., Nelson, D.E. & Young, K.D. Escherichia coli mutants lacking all possible combinations of eight penicillin binding proteins: viability, characteristics, and implications for peptidoglycan synthesis. *Journal of bacteriology* **181**, 3981-3993 (1999).
2. Lee, T.K. *et al.* A dynamically assembled cell wall synthesis machinery buffers cell growth. *Proceedings of the National Academy of Sciences of the United States of America* **111**, 4554-4559 (2014).
3. Datsenko, K.A. & Wanner, B.L. One-step inactivation of chromosomal genes in Escherichia coli K-12 using PCR products. *Proceedings of the National Academy of Sciences of the United States of America* **97**, 6640-6645 (2000).
4. Quan, S. *et al.* Adaptive evolution of the lactose utilization network in experimentally evolved populations of Escherichia coli. *PLoS Genet.* **8**, e1002444 (2012).
5. Philippe, N., Alcaraz, J.P., Coursange, E., Geiselmann, J. & Schneider, D. Improvement of pCVD442, a suicide plasmid for gene allele exchange in bacteria. *Plasmid* **51**, 246-255 (2004).
6. Baba, T. *et al.* Construction of Escherichia coli K-12 in-frame, single-gene knockout mutants: the Keio collection. *Molecular systems biology* **2**, 2006 0008 (2006).
